# Cardiovascular Disease (CVD) Risk in Women With Endometriosis: A Comprehensive Meta‐Analysis

**DOI:** 10.1002/clc.70443

**Published:** 2026-08-11

**Authors:** Maneeth Mylavarapu, Jeffry Samuel, Ravitej Thandi, Rizwan Mubashir, Vaishnavi Reddy Veeranagari, Aly Barakat, Joao Ferreira de Barros Neto, Alaa Osman, Sudeeshna Thiyagarajan, Reem Mohamed, Muhammad Junaid Hassan

**Affiliations:** ^1^ Department of Cardiology Endeavor Health Northshore Cardiovascular Institute, Endeavor Health Glenbrook Hospital Glenview Illinois USA; ^2^ Department of Medicine Division of Cardiology, University of Chicago Pritzker School of Medicine Chicago Illinois USA; ^3^ Department of Medicine Sugam Hospital Chennai Tamil Nadu India; ^4^ Department of Medicine Wenzhou Medical University Wenzhou Zhejiang China; ^5^ Department of Medicine North Cumbria Integrated Care NHS Foundation Trust Carlisle UK; ^6^ Department of Medicine Sri Ramachandra Institute of Higher Education and Research Chennai Tamil Nadu India; ^7^ Department of Internal Medicine Medway NHS Foundation Trust Kent UK; ^8^ Department of Medicine Rio de Janeiro State University (UERJ; Portuguese: Universidade do Estado do Rio de Janeiro) Rio de Janeiro Brazil; ^9^ Department of Medicine Lebanese University Faculty of Medical Sciences Hadath Lebanon; ^10^ Department of Medicine University Hospitals Coventry and Warwickshire NHS Trust Coventry UK; ^11^ Department of Medicine Hamad Medical Corporation Doha Qatar; ^12^ Department of Medicine Faisalabad Medical University Faisalabad Pakistan

**Keywords:** cardiovascular disease, coronary artery disease, endometriosis, major adverse cardiovascular events, meta‐analysis, systematic review

## Abstract

**Background:**

Cardiovascular disease (CVD) remains the leading cause of mortality globally. Emerging evidence indicates that cardiovascular risk in women is frequently underestimated, underscoring the importance of recognizing sex‐specific contributors. Endometriosis is a chronic inflammatory disorder affecting 5%−10% of reproductive‐aged women. However, previous syntheses evaluating its cardiovascular implications have yielded inconsistent results or were narrow in scope.

**Methods:**

A systematic literature search was conducted across databases, adhering to PRISMA guidelines. Studies comparing cardiovascular outcomes in women with versus without endometriosis were included. Primary outcomes were overall CVD and major adverse cardiovascular events (MACE). Secondary outcomes included ischemic heart disease (IHD), coronary artery disease (CAD), heart failure (HF), arrhythmias, coronary revascularization, and a composite of angina/acute myocardial infarction (Angina‐AMI). Random‐effects models with inverse variance weighting were used to calculate risk ratios (RRs) and 95% confidence intervals (CIs).

**Results:**

Women with endometriosis exhibited a significantly higher risk of primary outcomes, including overall CVD (RR 1.21; 95% CI 1.09–1.35; *p* = 0.004) and MACE (RR 1.23; 95% CI 1.10–1.38; *p* = 0.01). Regarding secondary outcomes, endometriosis was significantly associated with elevated risks of IHD (RR 1.57; 95% CI 1.14–2.17; *p* = 0.01), CAD (RR 1.36; 95% CI 1.32–1.41; *p* < 0.0001), and Angina‐AMI (RR 1.62; 95% CI 1.18–2.21; *p* = 0.002). Moderate‐to‐high statistical heterogeneity was observed across most secondary endpoints, except for CAD.

**Conclusion:**

Endometriosis is significantly associated with an increased risk of overall CVD, MACE, and ischemic coronary complications. These findings suggest that endometriosis should be recognized as an important sex‐specific modifier of cardiovascular risk.

## 1 Introduction

Cardiovascular diseases (CVD) continue to remain the leading cause of mortality globally, accounting for approximately one‐third of all deaths worldwide [1, 2]. Although recent decades have seen a steady decline in avoidable cardiovascular mortality within European Union nations, this reduction has been significantly more pronounced in men than in women [3]. Emerging evidence suggests that cardiovascular risk in female patients is frequently underestimated, directly contributing to clinical underdiagnosis, undertreatment, and a sharp relative increase in atherosclerotic cardiovascular disease (ASCVD) mortality among middle‐aged women between 45 and 64 years of age [3, 4, 5]. Beyond traditional risk factors such as hypertension, diabetes, dyslipidemia, early familial arterial disease, obesity, smoking, physical inactivity, and poor diet, recent epidemiological studies have identified several sex‐specific contributors to female cardiovascular risk. These unique risk factors include premature menopause, polycystic ovary syndrome (PCOS), migraines, primary ovarian insufficiency, and adverse pregnancy outcomes such as preeclampsia or gestational diabetes [6, 7, 8, 9, 10].

Among these sex‐specific conditions, endometriosis has emerged as a major public health concern with potentially profound cardiovascular implications. Endometriosis is a chronic, hormone‐dependent inflammatory disorder defined by the implantation of endometrial glands and stroma outside the uterine cavity [11]. The condition affects an estimated 5%−10% of women of reproductive age globally, which translates to roughly 176−190 million individuals [11, 12]. It frequently presents with debilitating clinical symptoms, including chronic pelvic pain, fatigue, congestive dysmenorrhea, heavy menstrual bleeding, severe dyspareunia, and infertility [13]. Managing the disease requires interdisciplinary, patient‐centered interventions involving long‐term hormonal therapies or surgical excision of lesions to mitigate its severe impact on overall quality of life [11, 14].

The underlying pathobiology of endometriosis shares several critical cellular and molecular mechanisms with the development of atherosclerotic coronary artery disease (CAD) [15]. Endometriosis drives both a localized and systemic chronic inflammatory state characterized by elevated oxidative stress, the accumulation of reactive oxygen species, endothelial dysfunction, and an atherogenic lipid profile [15, 16]. These interconnected pathways act as key mediators of vascular endothelial insult and atheromatous plaque progression, potentially accelerating the development of subclinical atherosclerosis and raising overall CVD risk [17].

Although multiple meta‐analyses [18, 19, 20, 21] have tried to highlight a potential link between endometriosis and heightened risk of cardiovascular outcomes, the findings have either been inconsistent or the studies were limited to a narrow range of outcomes. Furthermore, there exists a lack of consensus introducing a clinical uncertainty regarding the necessity and timing of routine cardiovascular screening in this patient population. Additionally, multiple expert consensus have underscored the need for comprehensive investigations in this landscape [22, 23].

To address these critical evidence gaps and clarify these discrepant findings, a rigorous, updated synthesis is essential. This review serves as a comprehensive meta‐analysis to evaluate the precise association between endometriosis and specific macrovascular complications, including CAD and ischemic heart disease (IHD). Ultimately, these findings aim to provide a more nuanced framework to guide risk stratification, clinical guidelines, and future research efforts in women's cardiovascular health.

## 2 Methods

### 2.1 Protocol and Registration

This systematic review and meta‐analysis were conducted in accordance with the Preferred Reporting Items for Systematic Reviews and Meta‐Analyses (PRISMA) guidelines [24].

### 2.2 Information Sources & Search Strategy

A comprehensive systematic literature search was conducted across PubMed/MEDLINE, Google Scholar, EMBASE, and Scopus from database inception through May 2026. The search strategy combined Medical Subject Headings (MeSH) terms and free‐text keywords related to “endometriosis,” “cardiovascular disease,” “coronary artery disease,” “arrhythmia,” “heart failure,” “myocardial infarction,” and “ischemic heart disease.” Reference lists of included studies and relevant systematic reviews were manually screened to identify additional eligible studies. A detailed search strategy was included in the Supporting Information S1: File S1.

### 2.3 Study Selection & Data Extraction

Titles and abstracts were initially screened for potential eligibility. Following that, full‐text articles of potentially relevant studies were retrieved and assessed against the predefined inclusion criteria (Supporting Information S1: File S2). Screening was performed by two independent reviewers (J.S. and V.R.V.), and disagreements between reviewers were resolved through consultation with a third reviewer (M.M.). Studies that reported cardiovascular outcomes in women with endometriosis compared to women without endometriosis were included.

Data were extracted by one of the authors (A.O.) using a standardized data extraction form, and reviewed by another (M.M.) to ensure validity of extraction. The following information was collected from each study: first author, publication year, study location, sample size, age, diagnosis of endometriosis, and cardiovascular outcomes.

### 2.4 Risk of Bias Assessment

The risk of bias (RoB) for most of the included studies was assessed using the Newcastle−Ottawa Scale (NOS) [25] for cohort studies. Each study was scrutinized across three major structural domains: the selection of the study cohorts (maximum of 4 stars), the baseline comparability of the cohorts to account for confounding (maximum of 2 stars), and the assessment of outcomes alongside follow‐up adequacy (maximum of 3 stars). The cumulative NOS scores ranged from 6 to 8 stars, reflecting a generally high methodological standard and a low‐to‐moderate risk of bias across the included literature.

### 2.5 Outcome Definition & Statistical Analysis

The primary outcomes include overall CVD and MACE (major adverse cardiovascular event), which includes acute myocardial infarction (AMI), stroke, and cardiovascular mortality [26].

Secondary outcomes include IHD, CAD, arrhythmias, new onset or incident heart failure (HF), composite of angiographically confirmed angina and AMI (Angina‐AMI), coronary revascularizations, that is, a composite of coronary artery bypass grafting (CABG) and percutaneous coronary intervention (PCI).

Meta‐analysis was performed using random‐effects models with inverse variance weighting to account for anticipated heterogeneity across studies [27]. Risk ratios (RR) with 95% confidence intervals (CIs) were calculated to understand the risk of a cardiovascular outcome in women with endometriosis. Heterogeneity was assessed using the Cochran's *Q* test and quantified using the *I* ^2^ statistic, with *I* ^2^ values of 25%, 50%, and 75% representing low, moderate, and high heterogeneity, respectively [28, 29]. All statistical analyses were conducted using the metaanalysisonline [30]. A *p* ≤ 0.05 was considered statistically significant.

## 3 Results

### 3.1 Study Selection Process

**Figure 1:**
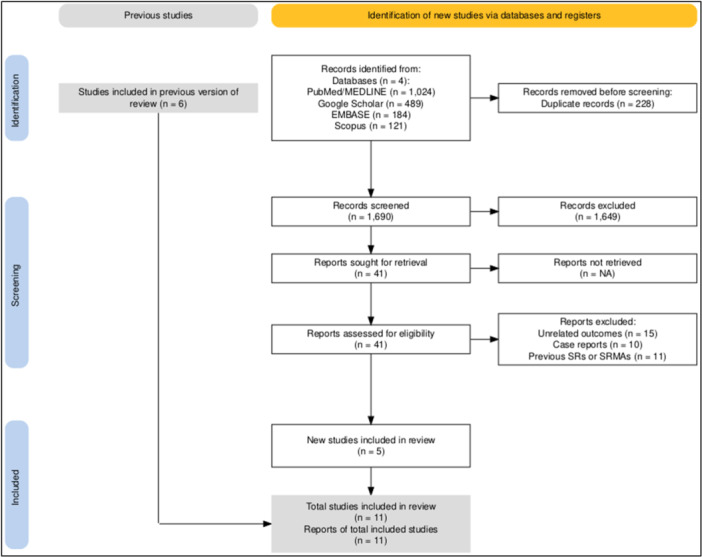
PRISMA Flowchart of study selection process.

A total of 1918 potential studies were identified across multiple databases and previous meta‐analyses. Of the 1918, 228 duplicates were removed, and 1649 were excluded after title and abstract (TiAb) screening, and 41 studies were reviewed by their full‐text. Ultimately, 11 studies were included. Figure 1 outlines the study selection process [31].

### 3.2 Baseline Characteristics

A total of 11 studies [32, 33, 34, 35, 36, 37, 38, 39, 40, 41, 42] were included. Of the 11, three were prospective cohort [36, 38, 40], two were retrospective cohort [33, 39], and six were retrospective databases/registries [32, 34, 35, 37, 41, 42]. Regarding geographical variations, five studies were from North America (three from the USA and two from Canada) [36, 37, 38, 40, 41], three were from Asia (three from Taiwan) [32, 34, 35], and three were from Europe (two from the UK and one from Denmark) [33, 39, 42]. Table 1 outlines the baseline characteristics of included studies.

**Table 1:** Baseline characteristics of included studies.

Study author & year	Study design & setting	Geographic location	Study tracking period	Cohort sample size (*n*)	Age
Blom et al. 2023	Retrospective population‐based matched cohort study	Canada	1993–2015 (Follow‐up to Dec 2017)	Endometriosis: 166 853 Controls: 333 706	Mean age: 36.4 ± 8.0 years
Bougie et al. 2025	Retrospective population‐based matched cohort study	Canada	1993–2015	Endometriosis: 166 853 Controls: 333 706	Mean age: 36.4 ± 8.0 years
Chiang et al. 2021	Retrospective population‐based cohort study	Taiwan	1997–2013	Endometriosis: 17 543 Controls: 70 172	Median age: 38 years (IQR: 31–44)
Havers‐Borgersen et al. 2024	Nationwide registry‐based observational cohort study	Denmark	1977–2021	Endometriosis: 60 508 Controls: 242 032	Median age: 37.3 years (IQR: 29.9–44.6)
Li et al. 2021	Retrospective population‐based cohort study	Taiwan	2000–2012 (Follow‐up to December 2013)	Endometriosis: 19 454 Controls: 77 816	Mean age: 37.4 ± 8.95 years
Mu et al. 2016	Large‐scale prospective longitudinal cohort study (NHS II)	United States	1989–2009 (Extended to 2015/2019 in offshoots)	Total evaluated cohort: 116 430 female nurses (endometriosis cases accrued over follow‐up)	Range at entry: 25–42 years
Okoth et al. 2021	Retrospective matched cohort study (THIN database)	United Kingdom	1995–2018	Endometriosis: 56 090 Controls: 223 669	Median age: 36.7 years (IQR: 30.9–42.5)
Powell et al. 2022	Retrospective genetic cohort sub‐study within NHS II	United States	1989–2015	Analyzed genotyped subset: 8170 participants (total sample: 106 831)	Mean age at entry: 34.4 ± 4.6 years
Wang et al. 2024	Prospective longitudinal cohort study (NHS II)	United States	1989–2019	Total evaluated cohort: 110 091 clean‐history women	Range at recruitment: 25–42 years
Wei et al. 2021	Retrospective population‐based propensity score‐matched cohort study	Taiwan	2000–2012 (Follow‐up to December 2013)	Endometriosis: 13 988 Controls: 13 988	Mean age: 37.8 ± 8.4 years
Meijs et al. 2026	Retrospective database (UK Biobank)	United Kingdom	Upto 2026	Endometriosis: 8101 Controls: 253 480	Median age: 57 years

### 3.3 Primary Outcomes

**Figure 2:**
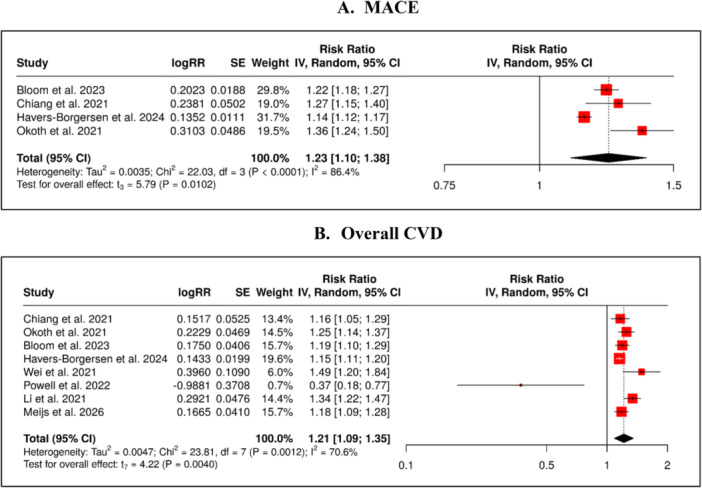
Primary outcomes: A. MACE and B. Overall CVD.

Women with endometriosis were associated with a significantly higher risk of overall CVD (RR 1.21; 95% CI 1.09−1.35; *p* = 0.004) compared to women without endometriosis. Furthermore, women with endometriosis were also associated with a significantly higher risk of MACE (RR 1.23; 95% CI 1.10−1.38; *p* = 0.01) compared to women without endometriosis (Figure 2).

### 3.4 Secondary Outcomes

**Figure 4:**
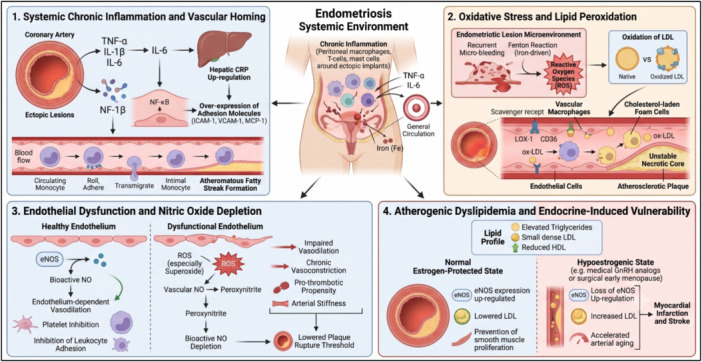
Mechanisms connecting endometriosis to cardiovascular disease.

Regarding secondary outcomes, women with endometriosis were associated with significantly higher risk of IHD (RR 1.57; 95% CI 1.14−2.17; *p* = 0.01), CAD (RR 1.36; 95% CI 1.32−1.41; *p* < 0.0001), Angina‐AMI (RR 1.62; 95% CI 1.18−2.21; *p* = 0.002) (Figure 4). However, no significance was found in regards to HF (1.13; 95% CI 0.88−1.43; *p* = 0.169), arrhythmias (RR 1.22; 95% CI 0.98−1.52; *p* = 0.06), and risk of coronary revascularization (RR 1.90; 95% CI 0.72−5.02; *p* = 0.20) (Figure 3).

Figure 3Secondary outcomes: A. IHD, B. CAD, C. Angina‐AMI, D. CABG/PCI, E. HF, F. Arrhythmias.

**Figure:**
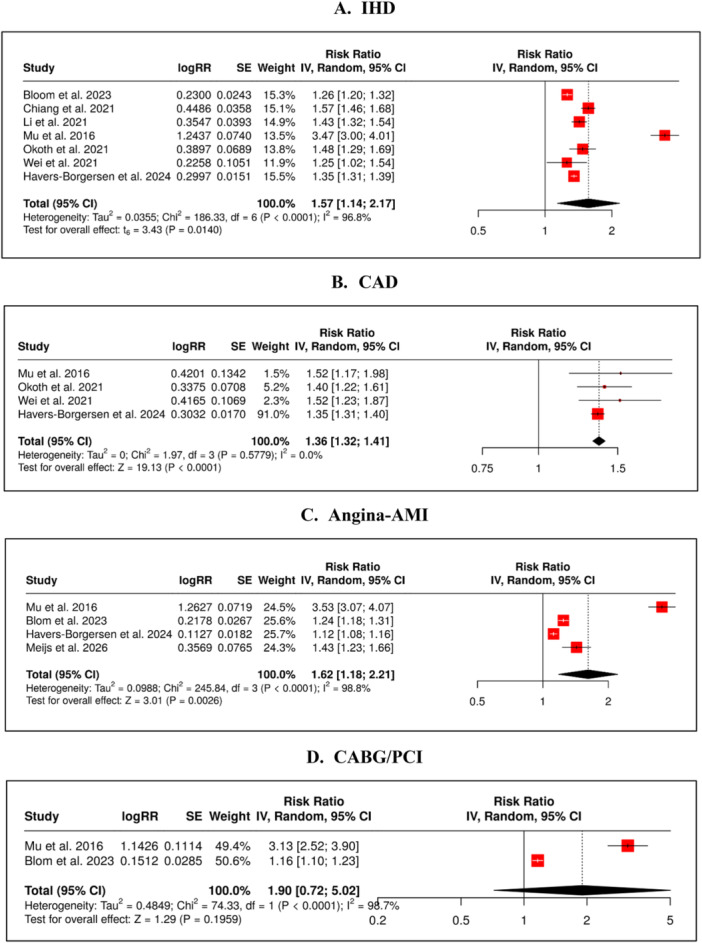

**Figure:**
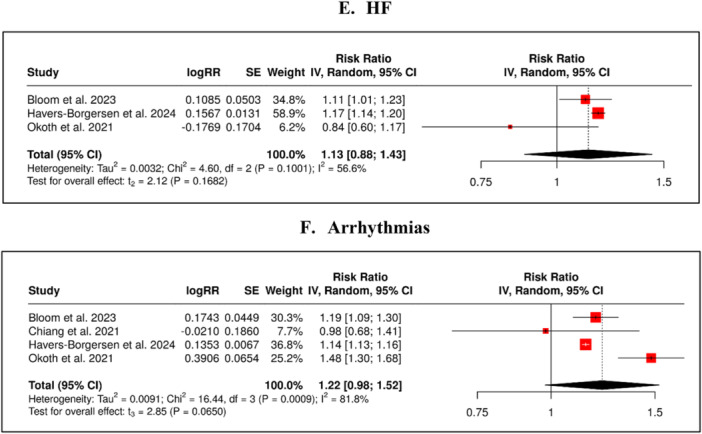

### 3.5 Risk of Bias Assessment

Across the cohort selection domain, eight studies (80.0%) demonstrated strong population representativeness by drawing participants from nationwide or regional registries. Crucially, all 10 studies established appropriate non‐exposed controls from identical source frameworks and successfully verified that the primary cardiovascular or mortality outcomes of interest were entirely absent at baseline. Independent blind assessments or linked administrative database registries were uniformly utilized to ascertain final outcomes over extended follow‐up intervals sufficient for tracking chronic disease progression. Supporting Information S1: File S3 outlines the methodological quality assessment of included studies.

### 3.6 Heterogeneity Assessment

Regarding heterogeneity assessment, CAD (*I* ^2^ = 0.0%, *p* = 0.58), demonstrating excellent consistency across the pooled study cohorts. Moderate‐to‐high heterogeneity was noted HF (*I* ^2^ = 56.6%, *p* = 0.10) and CVD (*I* ^2^ = 70.6%, *p* = 0.001). Conversely, high statistical heterogeneity (*I* ^2^ > 80%) was present in the remaining five endpoints, that is, Angina‐AMI (*I* ^2^ = 98.8%, *p* < 0.0001), CABG/PCI (*I* ^2^ = 98.7%, *p* < 0.0001), and IHD (*I* ^2^ = 96.8%, *p* < 0.0001). Supporting Information S1: Files S4 and S5 outline the heterogeneity assessment and publication bias, respectively.

## 4 Discussion

This comprehensive systematic review and meta‐analysis synthesizes the global evidence to establish the precise relationship between endometriosis and a broad spectrum of cardiovascular complications. By pooling data from 11 longitudinal cohorts and registries encompassing diverse geographic populations across North America, Europe, and Asia, our study provides robust clinical evidence that endometriosis is associated with heightened cardiovascular risk. Notably, our primary analysis demonstrates that women with endometriosis face a statistically significant 21% increase in the risk of overall CVD and a 23% increase in the risk of MACE. These findings underscore a critical paradigm shift in women's health: the clinical sequelae of endometriosis extend far beyond pelvic morbidity, directly predisposing patients to life‐threatening macrovascular events.

The stratified analysis of secondary endpoints adds crucial granularity to these findings, revealing that the overarching cardiovascular risk is heavily driven by atherosclerotic and ischemic phenotypes. We observed a striking 57% increased risk of IHD, a 36% increased risk of CAD, and a 62% increased risk of the composite Angina‐AMI endpoint. Interestingly, our analysis did not demonstrate a statistically significant correlation with incident HF, arrhythmias, or coronary revascularization procedures. The lack of significance for revascularization (CABG/PCI), despite the highly significant elevation in underlying CAD and ischemic events, may reflect historical patterns of clinical underestimation and conservative management of ischemic symptoms in female patients.

Our findings also align with a few of the previous meta‐analyses [21, 43] regarding increased risk of overall CVD risk, CAD, and MACE events. Furthermore, recent studies have shown that endometriosis is associated with early subclinical cardiac remodeling, largely mediated by metabolic and inflammatory pathways. Feng et al. [44] reported that CMR (cardiac magnetic resonance) imaging in women with endometriosis revealed subclinical concentric remodeling, including increased interventricular septal thickness, relative wall mass, and septal‐to‐lateral wall thickness ratio. In mediation analyses, metabolic dysfunction was identified as the primary pathway, followed by oxidative stress and inflammation. However, a Mendelian randomization study by Lu et al. [45] reported that a causal role of endometriosis in major CVD cannot be established.

### 4.1 Biological Plausibility and Pathophysiological Mechanisms

The pathophysiological mechanism linking endometriosis to macrovascular complications is primarily rooted in a shared chronic, systemic inflammatory state that drives endothelial dysfunction. Endothelial dysfunction represents one of the earliest detectable markers of arterial wall deterioration and subclinical atherosclerosis, occurring even in the absence of traditional cardiovascular risk factors. Recent vascular physiology studies demonstrate that women with endometriosis exhibit significantly impaired endothelial function, as evidenced by reduced flow‐mediated dilation, alongside greater advanced glycation end‐products skin accumulation and increased arterial stiffness [46, 47, 48]. This process is fueled by a profound local and systemic upregulation of pro‐inflammatory cytokines, oxidative stress markers, and reactive oxygen species, which collectively induce vascular endothelial insult and accelerate atheromatous plaque progression [49]. Notably, clinical data show that endothelial dysfunction partially regresses following the surgical excision of endometriotic lesions, further reinforcing the direct pathobiological link between active disease burden and vascular health [50]. Figure 4 outlines a mechanistic figure connecting endometriosis to CVD.

In addition to direct endothelial injury, endometriosis is strongly intertwined with an unfavorable cardiovascular risk profile. Prospective cohort data indicate that women with endometriosis face a significantly higher longitudinal risk of developing traditional metabolic derangements, including hypertension, hypercholesterolemia, hypertriglyceridemia, and metabolic syndrome [51, 52, 53, 54]. Interestingly, in women who conceive spontaneously, endometriosis is associated with an increased incidence of hypertensive disorders of pregnancy, including preeclampsia [55, 56], indicating an established driver of permanent, premature vascular remodeling.

### 4.2 Limitations and Future Directions

While this meta‐analysis provides highly powered, pooled estimates, the findings must be interpreted within the context of several methodological limitations. First, all 11 included studies were observational in nature, consisting of prospective and retrospective cohorts or administrative registries, which makes them inherently susceptible to unmeasured confounding. The structural variables used for statistical adjustment varied across the primary literature, and several studies lacked comprehensive adjustment for major traditional cardiovascular risk factors such as baseline hypertension, diabetes, and dyslipidemia. Second, a significant risk of misclassification bias exists due to heterogeneous diagnostic criteria across the cohorts. While some primary studies relied strictly on surgical and histopathological confirmation of endometriosis, others utilized clinical suspicion or administrative diagnostic codes. Clinical suspicion alone may introduce false positives by misclassifying patients with severe primary dysmenorrhea, while reliance on standard pelvic imaging can miss superficial peritoneal lesions, leading to underdiagnosis and the inadvertent inclusion of affected women within control cohorts.

Third, our study is bounded by the geographic distribution of the available data, which was strictly limited to North America, East Asia, and Western Europe, potentially restricting the generalizability of these findings to low‐ and middle‐income nations or other ethnic demographics. Additionally, the presence of high statistical heterogeneity (*I* ^2^ > 80%) across multiple primary and secondary endpoints, including Angina‐AMI and IHD, indicates substantial variation in study designs, follow‐up durations, and baseline patient profiles that could not be fully reconciled through random‐effects modeling alone. Finally, although our NOS assessment demonstrated a generally high methodological standard among the included literature, the fact that a subset of studies exhibited lower comparative quality underscores the need for caution when interpreting composite outcomes.

To address these limitations, future research must prioritize the execution of large‐scale, prospective cohort studies that integrate standardized, preferably surgically confirmed, definitions of endometriosis. These future investigations should meticulously track longitudinal cardiovascular outcomes while fully adjusting for both traditional metabolic risk profiles and emerging, female‐specific vascular mediators. Moreover, translational studies are required to explore whether targeted anti‐inflammatory therapies or specific surgical interventions can successfully arrest the progression of subclinical atherosclerosis in this patient population.

## 5 Conclusion

In conclusion, this systematic review and meta‐analysis establishes that endometriosis is associated with a significantly higher risk of CVD and adverse cardiovascular outcomes. These findings firmly position endometriosis as a critical, sex‐specific indicator of premature CVD. Early cardiovascular screening, rigorous risk factor modification, and close interdisciplinary collaboration between gynecologists and cardiologists are strongly warranted to mitigate long‐term macrovascular complications and optimize risk stratification frameworks in women diagnosed with this chronic inflammatory disorder.

## Author Contributions

Conceptualization: Maneeth Mylavarapu. Methodology: Maneeth Mylavarapu, Jeffry Samuel, Ravitej Thandi, Rizwan Mubashir, Vaishnavi Reddy Veeranagari, Alaa Osman, Sudeeshna Thiyagarajan, Rizwan Mubashir, Muhammad Junaid Hassan. Formal analysis and investigation: Maneeth Mylavarapu. Writing the original draft preparation: Maneeth Mylavarapu, Jeffry Samuel, Ravitej Thandi, Rizwan Mubashir, Vaishnavi Reddy Veeranagari, Aly Barakat, Joao Ferreira de Barros Neto, Alaa Osman, Sudeeshna Thiyagarajan, Rizwan Mubashir, Muhammad Junaid Hassan. Writing, review, and editing: Maneeth Mylavarapu, Jeffry Samuel, Ravitej Thandi, Rizwan Mubashir, Vaishnavi Reddy Veeranagari, Aly Barakat, Joao Ferreira de Barros Neto, Alaa Osman, Sudeeshna Thiyagarajan, Rizwan Mubashir, Muhammad Junaid Hassan. All authors reviewed the final draft and approved it for publication.

## Funding

The authors have nothing to report.

## Ethics Statement

Not applicable, given this is a systematic review and meta‐analysis of data extracted from previously published studies.

## Consent

Not applicable, given this is a systematic review and meta‐analysis of data extracted from previously published studies.

## Conflicts of Interest

The authors declare no conflicts of interest.

## Supporting information


Supporting File

## Data Availability

The data underlying this article are available in the article and its online supplementary material.
