# Supplementary material for: Cardiovascular Disease (CVD) Risk in Women With Endometriosis: A Comprehensive Meta‐Analysis
Source: Clin Cardiol. 2026 Aug 11;49(8):e70443. doi: 10.1002/clc.70443 (PMC13459647; doi:10.1002/clc.70443)
Supplement: Supplementary file 1 [file CLC-49-e70443-s001.docx]

**Supplementary Files**

**Supplementary File S1. Search Strategy**

| **Database** | **Search Fields / Restrictions** | **Optimized Search Syntax** |
| --- | --- | --- |
| **PubMed / MEDLINE** | Mesh Terms, Text Words, Title/Abstract ([Mesh], [Twin], [tiab]) | (("Endometriosis"[Mesh] OR "Endometriosis"[Twin] OR endometrioma*[tiab] OR endometriosis[tiab] OR adenomyosis[tiab]) AND ("Cardiovascular Diseases"[Mesh] OR "Coronary Artery Disease"[Mesh] OR "Myocardial Ischemia"[Mesh] OR "Heart Failure"[Mesh] OR "Arrhythmias, Cardiac"[Mesh] OR "Stroke"[Mesh] OR "Myocardial Infarction"[Mesh] OR "Cardiovascular Disease"[tiab] OR "CVD"[tiab] OR "Major Adverse Cardiovascular Events"[tiab] OR "MACE"[tiab] OR "Coronary Artery Disease"[tiab] OR "CAD"[tiab] OR "Ischemic Heart Disease"[tiab] OR "IHD"[tiab] OR "Coronary Heart Disease"[tiab] OR "CHD"[tiab] OR "Myocardial Infarction"[tiab] OR "Heart Attack"[tiab] OR "Stroke"[tiab] OR "Cerebrovascular Accident"[tiab] OR "Heart Failure"[tiab] OR "Arrhythmia*" OR "Atrial Fibrillation"[tiab] OR "Angina"[tiab] OR "Coronary Revascularization"[tiab] OR "PCI"[tiab] OR "CABG"[tiab])) |
| **Embase (Elsevier)** | Emtree Terms, Titles, Abstracts (/exp, :ti,ab) | ('endometriosis'/exp OR 'endometrioma':ti,ab OR 'endometriosis':ti,ab OR 'adenomyosis':ti,ab) AND ('cardiovascular disease'/exp OR 'coronary artery disease'/exp OR 'ischemic heart disease'/exp OR 'heart failure'/exp OR 'heart arrhythmia'/exp OR 'cerebrovascular accident'/exp OR 'major adverse cardiac event'/exp OR 'heart infarction'/exp OR 'cardiovascular disease':ti,ab OR 'cvd':ti,ab OR 'mace':ti,ab OR 'coronary artery disease':ti,ab OR 'cad':ti,ab OR 'ischemic heart disease':ti,ab OR 'ihd':ti,ab OR 'myocardial infarction':ti,ab OR 'heart failure':ti,ab OR 'arrhythmia':ti,ab OR 'angina':ti,ab OR 'stroke':ti,ab OR 'coronary revascularization':ti,ab OR 'pci':ti,ab OR 'cabg':ti,ab) |
| **Scopus (Elsevier)** | Title, Abstract, Index Keywords (TITLE-ABS-KEY) | ( TITLE-ABS-KEY ( endometrioma* OR endometriosis OR adenomyosis ) ) AND ( TITLE-ABS-KEY ( "cardiovascular disease*" OR "CVD" OR "major adverse cardiovascular events" OR "MACE" OR "coronary artery disease" OR "CAD" OR "ischemic heart disease" OR "IHD" OR "coronary heart disease" OR "CHD" OR "myocardial infarction" OR "heart attack" OR stroke OR "cerebrovascular accident" OR "heart failure" OR arrhythmia* OR "atrial fibrillation" OR angina OR "coronary revascularization" OR "PCI" OR "CABG" ) ) |
| **Google Scholar** | Title/Text Word Search (Simplified string for interface limits) | "endometriosis" AND ("cardiovascular disease" OR "coronary artery disease" OR "MACE" OR "stroke") |

**Supplementary File S2. Study Selection Criteria**

| **PECOS Domain** | **Inclusion Criteria (Eligible for Pooling)** | **Exclusion Criteria (Ineligible for Pooling)** |
| --- | --- | --- |
| **Population (P)** | Women of reproductive or postmenopausal age with a documented history or diagnosis of endometriosis. | Women with primary dysmenorrhea or pelvic pain syndromes lacking a verified or clinically coded diagnosis of endometriosis. |
| **Exposure (E)** | Endometriosis confirmed via surgical laparoscopy, histopathology, clinical visual metrics, or validated administrative billing codes (ICD-9/10/11). | No clear documentation of endometriosis status or exposure classification. |
| **Comparator (C)** | Women from identical source frameworks or cohorts with no lifetime history or clinical tracking of endometriosis (healthy controls or matched reference cohorts). | Studies without a distinct unexposed comparison cohort (e.g., single-arm cohorts). |
| **Outcomes (O)** | Primary: Overall CVD, MACE (AMI, stroke, CV mortality).Secondary: IHD, CAD, Arrhythmias, incident Heart Failure (HF), Angina-AMI composite, Coronary Revascularizations (PCI/CABG). | Studies tracking only subclinical imaging biomarkers (e.g., flow-mediated dilation, carotid intima-media thickness, magnetic resonance strain values) without collecting hard macrovascular event rates. |
| **Study Design (S)** | Longitudinal observational studies, including prospective cohorts, retrospective cohorts, and population-level administrative or genetic registry tracking databases. | Cross-sectional studies, case-control structures (prone to severe recall bias in chronic disease), case reports/series, experimental animal models, in vitro profiles, editorial pieces, or narrative reviews. |

**Supplementary File S3. Qualitative Assessment**

| **Study (Author, Year)** | **Representativeness of Exposed Cohort** | **Selection of Non-Exposed Cohort** | **Ascertainment of Exposure** | **Outcome Not Present at Start** | **Adjusts for Primary Risk Factors** | **Adjusts for Secondary Modifiers** | **Assessment of Outcome** | **Sufficiency of Follow-Up Length** | **Total Score** |
| --- | --- | --- | --- | --- | --- | --- | --- | --- | --- |
| **Mu, 2016** | ★ | ★ | ★ | ★ | ★ | ★ | ★ | ★ | 8/9 |
| **Chiang, 2021** | ★ | ★ | ★ | ★ | ★ | — | ★ | ★ | 7/9 |
| **Okoth, 2021** | ★ | ★ | — | ★ | ★ | — | ★ | ★ | 7/9 |
| **Wei, 2021** | ★ | ★ | ★ | ★ | ★ | ★ | ★ | ★ | 8/9 |
| **Li, 2021** | ★ | ★ | ★ | ★ | ★ | ★ | ★ | ★ | 8/9 |
| **Powell, 2022** | — | ★ | ★ | ★ | ★ | ★ | ★ | ★ | 7/9 |
| **Blom, 2023** | ★ | ★ | — | ★ | ★ | — | ★ | ★ | 7/9 |
| **Havers-Borgersen, 2024** | ★ | ★ | — | ★ | ★ | ★ | ★ | ★ | 7/9 |
| **Wang, 2024** | — | ★ | ★ | ★ | ★ | ★ | ★ | ★ | 8/9 |
| **Bougie, 2025** | ★ | ★ | — | ★ | ★ | — | ★ | ★ | 7/9 |

**Supplementary File S4. Heterogeneity Assessment**

| **Outcome / Analysis** | **Included Studies (N)** | **τ2** | **χ2 (Q-statistic)** | **Degrees of Freedom (df)** | **P-value (χ2)** | **I2 (%)** |
| --- | --- | --- | --- | --- | --- | --- |
| **CABG / PCI** | 2 | 0.4849 | 74.33 | 1 | <0.0001 | 98.70% |
| **Coronary Artery Disease (CAD)** | 4 | 0 | 1.97 | 3 | 0.5779 | 0.00% |
| **Cardiovascular Disease (CVD)** | 8 | 0.0047 | 23.81 | 7 | 0.0012 | 70.60% |
| **Heart Failure (HF)** | 3 | 0.0032 | 4.6 | 2 | 0.1001 | 56.60% |
| **Ischemic Heart Disease (IHD)** | 7 | 0.0355 | 186.33 | 6 | <0.0001 | 96.80% |
| **MACE** | 4 | 0.0035 | 22.03 | 3 | <0.0001 | 86.40% |
| **Angina / AMI** | 4 | 0.0988 | 245.84 | 3 | <0.0001 | 98.80% |
| **Arrhythmia** | 4 | 0.0091 | 16.44 | 3 | 0.0009 | 81.80% |

**Supplementary File S5. Publication Bias Assessment**

A. **Angina - AMI**

B. **Arrhythmias**

C. **CABG/PCI**

D. **CAD**

E. **CVD**

F. **HF**

G. **IHD**

H. **MACE**
